# Royal Hospital for Diseases of the Chest

**Published:** 1914-07-11

**Authors:** Stuart de la Rue

**Affiliations:** Chairman. Shenley Hill, Shenley, Herts


### THE EDITOR'S LETTER-BOX.

### Royal Hospital for Diseases of the Chest.

*To the Editor of* The Hospital.

Sir,—My attention has been called to a paragraph which lately appeared in the Press and commented upon the opening at Oxford of an auxiliary collecting branch of the Royal Hospital for Diseases of the Chest.

I should, as chairman of the hospital, like to be allowed to state that this movement, although started in the hundredth year of the hospital's existence, is not intended to form part of the centenary campaign now being conducted, but is destined to be a continuing work.

The originators at Oxford had only a month in which to mature their plans prior to the actual collection, so that the first collection, accomplished in a week and yielding in the aggregate more than £30, mostly in small sums, is satisfactory.

The object of the movement, however, is not so much the collection of hard cash as the infusion of an interest in hospitals and their work into the coming generation; that hospitals may continue to draw their lay managers from that class which is best fitted to maintain and uphold the voluntary principle in hospitals so essential to the welfare of the patients.

Any Oxford undergraduates interested in this movement communicating with W. Fothergill Robinson, Esq., of Bishop King's Palace, Oxford, will gladly be furnished with full information.—Yours, etc.,

Stuart de la Rue, Chairman.

Shenley Hill, Shenley, Herts, July 4, 1914.

[As will be seen from our comments elsewhere in this issue (p. 402), we fully appreciate and sympathise with the objects which Mr. de la Rue avows.—Editor, The Hospital.]

